# CAENORHABDITIS ELEGANS AS A MODEL OF AIR POLLUTION TOXICITY DURING DEVELOPMENT AND LIFESPAN

**DOI:** 10.1093/geroni/igz038.366

**Published:** 2019-11-08

**Authors:** Amin Haghani, Hans M Dalton, Nikoo Safi, Farimah Shirmohammadi, Constantinos Sioutas, Todd E Morgan, Caleb E Finch, Sean P Curran

**Affiliations:** 1 Leonard Davis School of Gerontology, University of Southern California, Los Angeles, California, United States; 2 Center for Cancer Prevention and Translational Genomics at the Samuel Oschin Comprehensive Cancer Institute, Cedars-Sinai Medical Center, Los Angeles, California, United States; 3 Viterbi School of Engineering, University of Southern California, Los Angeles, California, United States

## Abstract

Air pollution (AirPoll) is among the leading human mortality risk factors and yet little is known about the molecular mechanisms of this global environmental toxin. Our recent studies using mouse models even showed genetic variation and sex can alter biological responses to air pollution. To expand genetic studies of AirPoll toxicity throughout the lifespan, we introduced Caenorhabditis elegans as a new AirPoll exposure model which has a short lifespan, high throughput capabilities and shared longevity pathways with mammals. Acute exposure of C. elegans to airborne nanosized AirPoll matter (nPM) caused similar gene expression changes to our prior findings in cell culture and mouse models. Initial C. elegans responses to nPM included antioxidant, inflammatory and Alzheimer homolog genes. The magnitude of changes was dependent on the developmental stage of the worms. Even short term exposure of C. elegans to nPM altered developmental and lifespan hormetic effects, with pathways that included skn-1/Nrf family antioxidant responses. We propose C. elegans as a new and complementary model for mouse and cultured cells to study AirPoll across the lifespan. Future chronic nPM exposure and high throughput genetic screening of C. elegans can identify other major regulators of the developmental and lifespan effects of air pollution. This work was supported by grants R01AG051521 (CEF); R21AG05020 (CEF); Cure Alzheimer’s Fund (CEF); R01GM109028 (SPC), F31AG051382 (HMD) and T32AG000037 (HMD), T32AG052374 (AH).

